# Open Cancer

**Published:** 1869-02

**Authors:** 


Open Cancer—We learn that the exqusite pain which belongs to open cancer is found to be best relieved by the stramonium ointment, which is employed in London. The following is a formula. Half a pound of fresh stramonium leaves, and two pounds of lard. Mix the bruised leaves with the lard, and expose to a mild heat until the leaves become friable, and strain through lint. The ointment thus prepared is spread upon lint, and the dressing changed three times a day.—*Medical and Surgical Reporter.*

